# Universes within universes: microbiome diversity associated with different body parts of the sand lizard (*Lacerta agilis*)

**DOI:** 10.7717/peerj.21061

**Published:** 2026-05-01

**Authors:** Joanna Michalska-Madej, Katarzyna Janik-Superson, Bartłomiej Zając, Mariusz Krupiński, Carl Smith, Michal Seweryn, Alejandro Ibáñez

**Affiliations:** 1 BioMedChem Doctoral School of the University of Lodz and Lodz Institutes of the Polish Academy of Science, University of Lodz, Łódź, Łódź voivodeship, Poland; 2 Department of Ecology and Vertebrate Zoology/Faculty of Biology and Environmental Protection, University of Lodz, Łódź, Łódź voivodeship, Poland; 3 Centre for Digital Biology and Biomedical Science—Biobank Lodz®/Faculty of Biology and Environmental Protection, University of Lodz, Łódź, Łódź voivodeship, Poland; 4 Department of Comparative Anatomy/Institute of Zoology and Biomedical Research, Jagiellonian University Cracow, Kraków, Lesser Poland voivodeship, Poland; 5 Department of Industrial Microbiology and Biotechnology/Faculty of Biology and Environmental Protection, University of Lodz, Łódź, Łódź voivodeship, Poland

**Keywords:** Body part, Core microbiome, *Lacerta agilis*, Microbial diversity, 16s rRNA, Next-generation sequencing

## Abstract

The bodies of animals host millions of microbial communities collectively known as the microbiome. The microbiome plays a crucial role in various processes related to the host’s health and well-being. Although our understanding of the microbiome’s importance to host functioning is growing rapidly, many aspects remain poorly understood. One such aspect is the role of the microbiome in chemical communication. To address this question, we used the sand lizard (*Lacerta agilis*), a reptile with well-developed chemosensory abilities and commonly distributed in Central Europe. Our first goal was to characterize the bacterial microbiome associated with different body parts potentially involved in chemical signalling (*e.g.*, femoral glands, cloaca, and skin). Additionally, we examined sex-related differences in the microbiome that could be connected to intraspecific communication. Over two years, a total of 274 samples were collected. Amplicon sequencing of the 16S rRNA V3–V4 region revealed significant variation in microbial diversity across body parts, with the skin hosting the most diverse and balanced communities. In contrast, the cloaca and femoral glands contained less diverse but more specialised assemblages. No differences in microbial diversity between sexes were observed, but the year of sampling was an important factor, suggesting a highly dynamic microbiome in sand lizards. There was minimal overlap in the number of unique operational taxonomic units (OTUs) between body parts, indicating a small core microbiome (∼1% of shared taxa). Sex differences in tissue-specific bacteria were more pronounced in the cloaca, supporting the idea that the cloacal microbiome is highly specialised. Our findings suggest that microbial communities vary significantly among body parts, with strong tissue specificity, indicating that each region provides a distinct ecological niche. This study offers promising directions for future research into how host-associated microbiomes could influence chemical communication in vertebrates.

## Introduction

The body of every animal is home to a wide variety of symbiotic microbial communities that together constitute the microbiome of the host organism. The microbiome plays an essential role in multiple processes related to the host’s health and well-being. Indeed, the microbiome is essential for animal survival (protecting the body against pathogenic microorganisms), influencing digestion, immunity, behaviour, and adaptation to changing environments (Turnbaugh & Gordon, 2009; Ross, Rodrigues Hoffmann & Neufeld, 2019). Understanding microbiome interactions can lead to advancements in medicine, conservation, and biotechnology. Although our knowledge about the relevance of the microbiome to the functioning of the host is quickly expanding, there is a lot still to discover, especially in wild populations of non-model vertebrates.

Recent research has underscored the role of microbiota in animal chemical communication. Even though research on this topic is still limited, some groups of vertebrates, such as mammals, have received more attention. Mammals possess scent glands over the body that are populated with dense and diverse symbiotic bacterial communities that vary with sex, reproductive state or social traits relevant for mating (Theis, Schmidt & Holekamp, 2012; Theis et al., 2013; Leclaire et al., 2017; Greene et al., 2019; Rojas et al., 2020). Unfortunately, other groups of vertebrates have been investigated even less than mammals, but there are few studies on the subject. For instance, in birds, it has been suggested that bacteria abundantly populate several structures that could emit odours, such as feathers and the uropygial gland (Maraci, Engel & Caspers, 2018). In amphibians, a close link between skin microbiome and scent production has been demonstrated by Brunetti et al. (2019). Thus, previous research indicates the potential involvement of microbes in chemo-signalling across major vertebrate groups.

Reptiles are a speciose class of vertebrates heavily relying on chemosignals for searching and choosing mates and for intrasexual interactions (Martín & López, 2024). Sand lizards (*Lacerta agilis*) are one of the most widespread reptiles in Eurasia and most common species in Central Europe with locally dense populations. Wild populations of sand lizards have been studied for decades, and their mating system is well understood. Females often mate multiple times, either with the same male or with several different males. After copulation, males temporarily guard the female with whom they have mated to reduce sperm competition and increase their chances of siring offspring in the clutch (Blanke & Fearnley, 2015). One of the main organs producing chemical signals present in many species of lizards—including *L. agilis*—are femoral glands (Mayerl, Baeckens & Van Damme, 2015; García-Roa et al., 2017). In sand lizards, femoral glands are dimorphic, being large and prominent in males, while females have small glands that are likely functionless. Laboratory experiments have shown that females tend to prefer odour cues secreted by femoral glands from males that are genetically more distant, likely as a mechanism to avoid inbreeding with close relatives (Olsson et al., 2003). Therefore, sand lizards are an ideal model to study chemical communication, including its relationship with the microbiome.

Besides femoral glands, other parts of the body, such as the skin, may emit chemosignals in lizards (Martín & López, 2024). Studies on skin microbiome are scarce in reptiles and mainly focused on the effect of pathogenic microorganisms, as well as the negative effects of these pathogens on health status (Ross, Rodrigues Hoffmann & Neufeld, 2019). Although currently only a few studies have examined the reptilian skin microbiome in an ecological context, a high diversity of microbes in the skin has been documented. For example, in brown spotted pit vipers (*Protobothrops mucrosquamatus*), the skin had a higher alpha microbial diversity than that of the oral cavity (Su et al., 2023). Similarly, in captive Komodo dragons, more diverse microbial communities were harboured by the skin compared to saliva or faeces (Hyde et al., 2016). Apart from the skin and femoral glands, the cloaca is another organ that might be involved in the production of chemical signals (Martín & López, 2024). The cloaca is particularly informative in terms of microbiota since it acts as a convergence point of the gastrointestinal, urinary, and reproductive tracts and serves as a potential medium for microbial exchange during copulation, oviposition, or defecation. Previous research shows that the cloacal microbiome is shaped by sex, reproductive season, and body size, as observed in stripped plateau lizards, *Sceloporus virgatus* (Bunker, Arnold & Weiss, 2022). Differences also occur between closely related species and populations, highlighting the influence of host relatedness and environmental similarity (Bunker, Arnold & Weiss, 2022). Moreover, cloacal samples differ markedly from other tissues—*e.g.*, intestine, faeces, or reproductive organs (Bunker, Martin & Weiss, 2022; Bunker et al., 2021). Thus, microbial communities may change across the body, underscoring the potential role of specific microbiota associated with distinct tissues on chemical communication.

The first aim of this study was to provide a comprehensive characterization of the microbiome of the sand lizard, exploring how the microbiome varies between different areas of the body. Since different body parts represent totally distinct micro-environments, we expected obvious differences in the microbiome. For instance, the skin is in direct contact with the ground, and it is likely to pick up bacteria from external sources. In contrast, colonization by external bacteria is less likely in other organs such as the cloaca or femoral glands. In addition, some body parts offer a more selective set of environmental parameters promoting the apparition of specialized microbes. For instance, epidermal glands—such as human sebaceous glands—offer lipid-rich anoxic conditions that can favour or inhibit the growth of specific microbes (Byrd, Belkaid & Segre, 2018). Another important aspect of this study was to examine sexual differences in the microbiome since it could be related to communicative processes between males and females during mating season. For that, we focused on sexual variation in shared and unique microbial communities across body parts. We expected little similarity in the microbial communities harboured by males and females. Given that chemical signals are typically produced by males (Mayerl, Baeckens & Van Damme, 2015), we predicted that males should have a rich and distinctive microbiome that could enhance pheromone production. In contrast, we did not predict that the bacterial community of females would play a role in chemical signaling and thus microbial communities should be less diverse and more homogeneous when compared to males.

## Material & Methods

### Microbiome sampling

**Figure 1:**
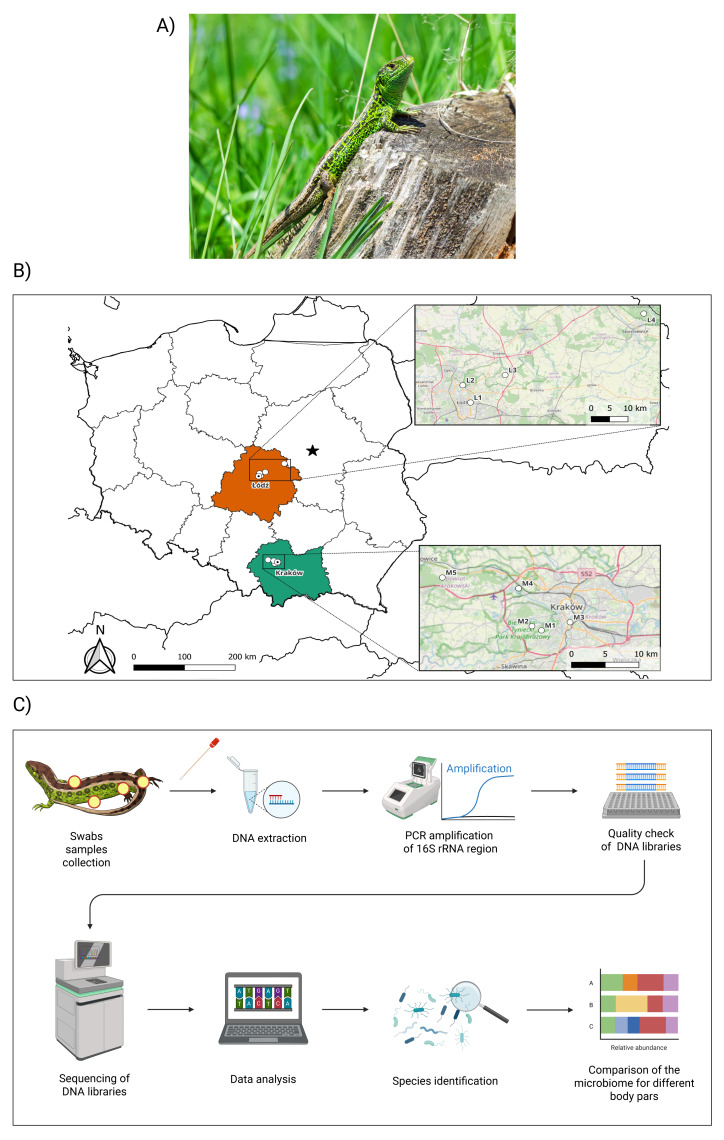
Main workflow of the study including locations. (A) Photograph of a sand lizard (*Lacerta agilis*) in its habitat. (B) An administrative map of Poland showing the two main regions located in the south (marked in green colour—Lesser Poland voivodeship) and the centre (marked in orange colour—Łódź voivodeship) of the country in which the study was conducted. The points represent the sampling sites, abbreviations according to Table 1. The map was drawn with QGIS. (C) Workflow of the study showing steps taken, including sampling in the field as well as laboratory and data analysis. Created in BioRender.

A total of 274 sand lizard samples were collected during the spring-early summer of the years 2022 and 2023 (see Table 1). Samples originated from 80 different lizards (plus four recaptures) collected at different locations from southern (Lesser Poland voivodeship) and central (Łódź voivodeship) Poland (see Figs. 1A, 1B and Table 1). Lizards were sexed according to external features (*i.e.,* males present an obvious bulge at the base of the tail where the hemipenes are stored, well-developed femoral glands, large head, and green nuptial colouration). A few individuals were categorized as juveniles when the sex could not be reliably assigned. The microbiome of lizards was sampled by using sterile dry swabs. Lizards were gently rinsed with Milli-Q water before sampling (this step was performed to remove superficial contaminants from the body surface, such as soil particles or plant debris). Afterwards, samples were taken from each lizard (at least one sample for each individual). Four body parts (dorsal skin, ventral skin, femoral glands, and cloacal region) were sampled by rubbing a sterile swab on each part. In the case of femoral glands, these were gently pressed to obtain secretions before rubbing the swabs on the surface of the glands. Femoral gland secretions were typically observed in males, but the same procedure was followed as described before in all individuals. After sampling, each swab was placed in a separate tube previously filled with ATL buffer. Samples were refrigerated during fieldwork in a portable cooler and subsequently stored at 4 °C until laboratory analysis. In addition, negative control tubes (seven samples) were collected from the air. Sterile swabs moistened with Milli-Q water were hold and gently waived in the air for approximately 10 s at the sites where lizards were captured. All environmental negative control samples were processed in the same way as the samples collected from the lizards, including DNA extraction, preparation of 16S rRNA amplicon libraries, sequencing, and downstream data analysis.

**Table 1:** Overview of the samples. Number of samples collected for each body part by year and sex (*i.e.*, male, female and juvenile/unsexed). Abbreviation for the sampling sites are shown as indicated in Fig. 1.

** **	**Year 2022**				**Year 2023**		
	Male	Female	Juvenile		Male	Female	Juvenile
**Body part**							
Dorsal skin	6	12	2		30	14	
Ventral Skin	3	10	2		33	16	
Femoral glands	9	15	3		29	15	
Cloacal region	9	12	1		36	17	
**Sampling location/Map**							
Młynka (Lesser Poland v.)**/M5**	6	25			12	21	
Kraków - Krzemionki (Lesser Poland v.)**/M3**	1						
Skierniewice (Łódź v.)**/L4**	3	8					
Łódź - 3rd May Park (Łódź v.)**/L1**	3	2	3		22	11	
Łódź - Arturówek (Łódź v.)**/L2**	5	8	1				
Boginia (Łódź v.)**/L3**	9	2					
Kraków - Campus UJ (Lesser Poland v.)**/M1**		4	4				
Kraków - Bodzów (Lesser Poland v.)**/M2**					70	29	
Kraków - Mydlniki (Lesser Poland v.)**/M4**					24	1	

This study was performed under permission from the Local Ethics Committee (39/ŁB 242/2022), the Directorate for Environmental Protection in Łódź (WPN.6401.10.2022.BWo) and the General Environmental Protection Directorate, Poland (DZP-WG.6401.116.2022.TŁ). Collection of body swabs for microbiome analysis is a non-invasive and harmless method. After sampling all lizards were released at the same capture sites.

### Laboratory work

Microbial DNA was extracted from swabs using the DNeasy PowerSoil Pro Kit (QIAGEN N.V., Hilden, Germany) following the manufacturer’s instructions. To check for reagent contamination, each part of the isolation contained a control sample (containing only ATL buffer), which was processed alongside the test samples. Some of the samples could not be used for library preparation because of insufficient DNA concentration.

Next-generation sequencing (NGS) of the 16S rRNA bacterial gene amplicon focused on the V3–V4 region. Amplicon libraries were prepared using the 16S Metagenomic Sequencing Library Preparation protocol, preparing 16S Ribosomal RNA Gene Amplicons for the Illumina MiSeq System (Illumina, San Diego, CA, USA). The initial steps in library preparation were repeated three times to minimize human handling errors. The primer sequences and polymerase chain reaction (PCR) reaction conditions are provided in Klindworth et al. (2013), Table S1. The positive control for the V3–V4 region of the 16S rRNA gene consisted of DNA isolated from the human saliva microbiome. PCR-grade water served as the negative control for the reaction. The resulting amplicons were purified using magnetic beads (AMPure XP beads; Beckman Coulter, Brea, CA, USA), following the Illumina^®^ protocol. Before pooling the samples, the total band volume was measured for each amplicon using the Gel Doc™ EZ Imaging System with Image Lab™ Software (Bio-Rad, Hercules, CA, USA). Samples were then diluted with PCR-grade water to achieve uniform concentrations and pooled. The final library concentration was determined according to the NEBNext Ultra DNA Library Prep Kit for Illumina (New England Biolabs^®^ Inc., Ipswich, MA, USA) protocol, and the pooled libraries were adjusted to a final concentration of 0.5 nM for sequencing. The libraries were sequenced on the Illumina NovaSeq 6000 platform with 2  × 250 bp paired-end reads. PhiX libraries (PhiX Control Kit v3, Illumina^®^) were included in the run as an internal positive quality control. Sequencing of the samples was performed at the Biobank Laboratory of the University of Lodz (Dobrowolska et al., 2019). The workflow followed in this study is shown in Fig. 1C.

### Data processing and microbial identification

Sequencing data was demultiplexed utilizing BCL2Fastq software (v2.19.0.316). Following this, adapters were excised with the Trim Galore software (v0.6.10). The DADA2 algorithm (implemented in QIIME2 v2024.2) was employed for the denoising procedure, encompassing quality filtering at a threshold greater than Q20, denoising, the merging of paired reads, and the elimination of chimeric reads. The trimming parameters employed were: trim forward -15, trim reverse -15, trunc forward -240, trunc reverse -240. In total, sequencing results were obtained for 281 samples, including 258 unique samples, seven controls, and 16 technical replicates.

Downsampling of the samples was performed to a sequencing depth of 100,000 reads. This operation was repeated 1,000 times to ensure reproducibility of the subsampling process, and the average abundance of each amplicon sequence variant (ASV) was calculated. ASVs were defined by grouping sequences (reads) with complete (100%) sequence similarity. Subsequently, operational taxonomic units (OTUs) were delineated at a 99% similarity threshold. To eliminate rare reads, the following algorithm was applied: the natural logarithm of the sum of occurrences of a given OTU in the samples was plotted on a density graph, and OTUs with values smaller than the first local minimum were removed (see Fig. S1).

To obtain a more accurate taxonomic classification of the OTU sequences, sequence alignments were performed using the standalone version of the blastn software (Nucleotide-Nucleotide BLAST 2.12.0+), executed *via* the command-line interface. The NCBI nucleotide (nt) database, updated as of October 2023, was downloaded and queried locally on a high-performance server at the Biobank Laboratory (University of Lodz) to ensure full control and reproducibility of the alignment process, avoiding reliance on the NCBI web-based interface or QIIME2 plugins. The output file generated from the blastn alignment for each OTU was used to extract taxonomic identifiers (taxonomic identifier—the unique NCBI identifier for the source organism). These identifiers were then processed using a locally downloaded version of the UniProt database (downloaded on 2025-05-11) to retrieve the corresponding taxonomic lineages (taxonomic lineage—the taxonomic classification of the source organism). To reduce the number of hits per OTU and increase classification specificity, filtering was applied using a custom script written in GNU Awk 5.1.0, API: 3.0 (GNU MPFR 4.1.0, GNU MP 6.2.1). The filtering criteria included: selection of hits with the lowest e-value, sequence identity ≥ 99%, and alignment length ≥ 400 nucleotides. The 99% identity threshold ensured highly specific matches; the 400 nt length cutoff allowed the exclusion of short, less informative sequences; and the lowest e-value reduced the chance of random matches, improving assignment confidence.

In the second step, to assign a taxonomic lineage to each OTU, an additional classification criterion was applied. If at least 60% of hits had the same taxonomic lineage, the result was accepted as valid. However, when an OTU did not meet the classification criteria, it was then considered “under threshold”. This criterion was applied at the *Family* level, excluding the *Genus* and *Species* levels. The 60% consensus threshold was selected as optimal based on preliminary analysis using a subset of the data (see Table S2).

### Statistical analysis

Data analysis was undertaken using the R statistical software, version 4.5.0 (R Core Team, 2025). For analysis, OTUs that appeared in less than five samples were dropped. Taxa classified as “Other Kingdom” and “environmental samples” (*e.g.*, see Fig. S2) were also filtered out of the analysis. Bacteria classified as “under threshold” were not filtered from the dataset and were used in the microbial diversity calculations and other analysis. The number of reads assigned to each taxon was standardized for the library size, and these values were used as a continuous variable. Then, values were rarefied using the function *rarefy_even_depth* (package *phyloseq*) to 90% of the minimum sample depth in the dataset. Bar plots were used to visualize microbiome composition using the 15 most abundant OTUs.

Venn diagrams were used to visualize the number of shared and unique OTUs across body parts to determine: (1) the existence of a core microbiome and (2) tissue-specific bacteria. In addition, we were interested in examining sexual patterns of variation in tissue-specific bacteria. For this purpose, differences between males and females in the number of shared *vs* unique OTUs for each body part were tested using Fisher’s exact tests.

Microbial alpha diversity (Observed and Shannon diversity) was calculated using the *estimate_richness* function in the *phyloseq* package. Variation in microbial diversity was modelled as a function of body part, sex and year. Because lizards were collected from multiple locations and included recaptured individuals and potentially multiple samples per lizard, data were modelled using a general linear mixed model (GLMM), with individual nested within location included as a random term.

Statistical analyses were done using a dataset that included only samples originating from sand lizards (Dataset-1). In addition, we used a second dataset (Dataset-2) that included sand lizard samples as well as seven control samples. The dataset was processed and filtered exactly as described above. Microbiome composition was visualized with the 15 most abundant OTUs showing obvious differences between control and sand lizard samples (see Fig. S2).

## Results

### Sequence summary and microbial identification

In total, 31,988 OTUs were identified across all samples. After sequence blasting, OTUs were classified in different categories. The largest number of OTUs were assigned to the Kingdom Bacteria (17,939 OTUs), including 2,963 OTUs that did not meet the classification criteria (classed as “under threshold”), and 86 OTUs with no taxonomy (*i.e.,* assigned by BLASTn as “no taxonomy”). In addition, 13,963 OTUs were classified to other kingdoms (*i.e.,* assigned by BLASTn to “Other Kingdom”).

Further classification was conducted based on the proportion of OTUs in each category. We presented graphically the proportion of bacterial phyla and main categories but excluded sequences classified to other kingdom for a better visualization (Fig. S2). The most abundant categories were “under threshold” (33.34%), environmental samples (22.31%), Pseudomonadota (21.63%), and Actinomycetota (14.48%). Other phyla, such as Bacteroidota (3.14%) and Bacillota (2.52%), were poorly represented in samples (Fig. S2).

### Microbiome composition

**Figure 2:**
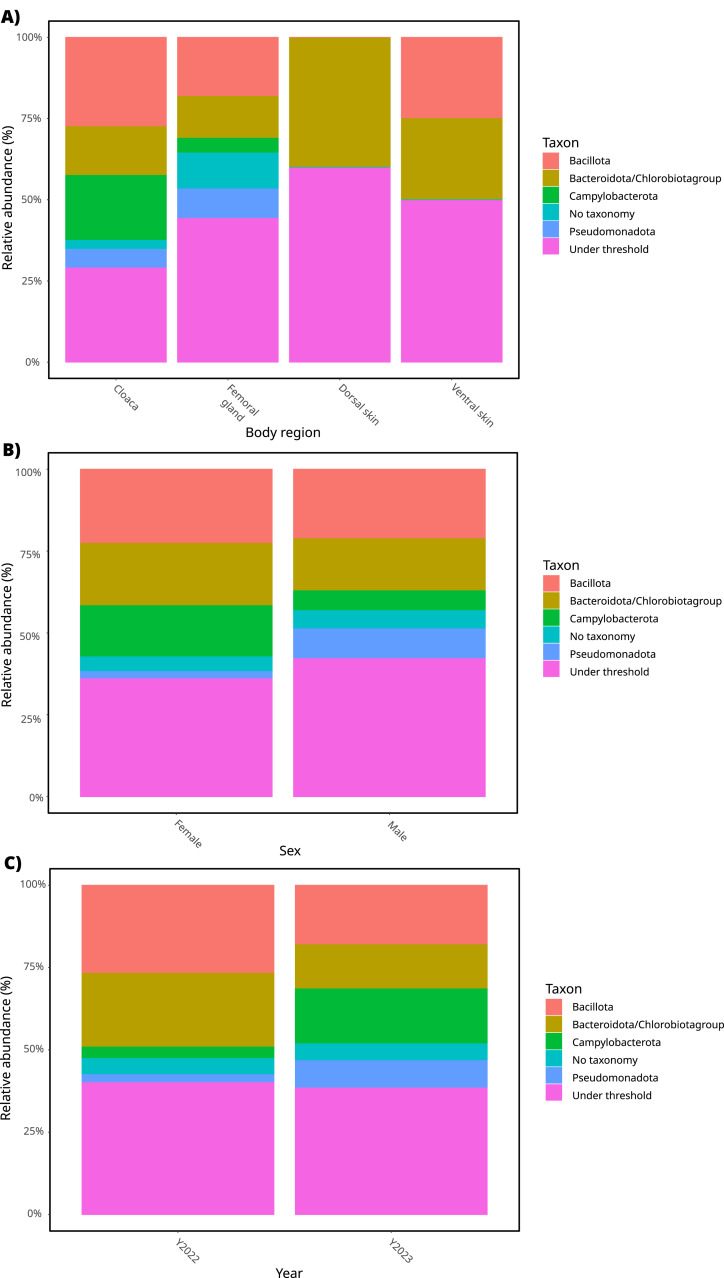
Composition of most abundant phyla. Most abundant phyla (top 15 operational taxonomic units, from Dataset-1) for sand lizard microbiome, including different factors considered in this study: (A) body part; (B) sex; (C) year.

After data filtering and processing (see ‘Materials and Methods’), we focused on the 15 most abundant OTUs and grouped those according to phyla. Microbiome composition changed sharply depending on the body part (Fig. 2A). A visual comparison indicated that Bacillota, Campylobacterota, Pseudommonadota, and Bacteroidota were the most abundant phyla in the cloaca and femoral glands (Fig. 2A). In contrast, dorsal and ventral skin were dominated by Bacteroidota and/or Bacillota (Fig. 2A). Notably, a substantial portion of the most abundant bacteria were assigned to the category “under threshold” in all analyzed samples. The inclusion of control samples in the dataset yielded generally similar results for the 15 most abundant OTUs in the cloaca and femoral glands. However, more pronounced differences in the abundance of Pseudomonadota were observed in the dorsal and ventral skin when compared to the dataset without control samples (Fig. S3). As expected, control samples showed an entirely different composition in comparison with sand lizard samples (Fig. S3).

Phyla composition was similar between sexes, with small variations only obvious in just some taxa (Fig. 2B). Pseudomonadota was slightly more abundant in males than in females (Fig. 2B). In contrast, Campylobacterota and Bacteroidota/Chlorobiota group were more abundant in females (Fig. 2B). An important portion of bacteria fell under the category “under threshold” for both sexes; this category was slightly more prominent in males than females (see Fig. 2B).

The composition of the most abundant bacteria was qualitatively similar between years (Fig. 2C). A higher abundance for Campylobacterota was observed in the year 2023. In contrast, Bacteroidota/Chlorobiota group was more abundant in the year 2022 than in 2023 (Fig. 2C).

### Microbial diversity

**Figure 3:**
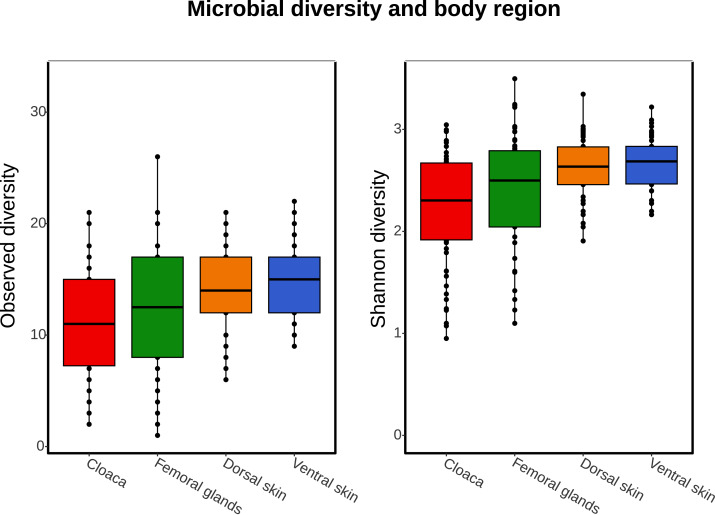
Data distribution for alpha diversity. Boxplots showing data distribution for microbial alpha-diversity metrics (Observed diversity and Shannon diversity) for each body part (cloaca, femoral glands, dorsal skin, and ventral skin).

**Figure 4:**
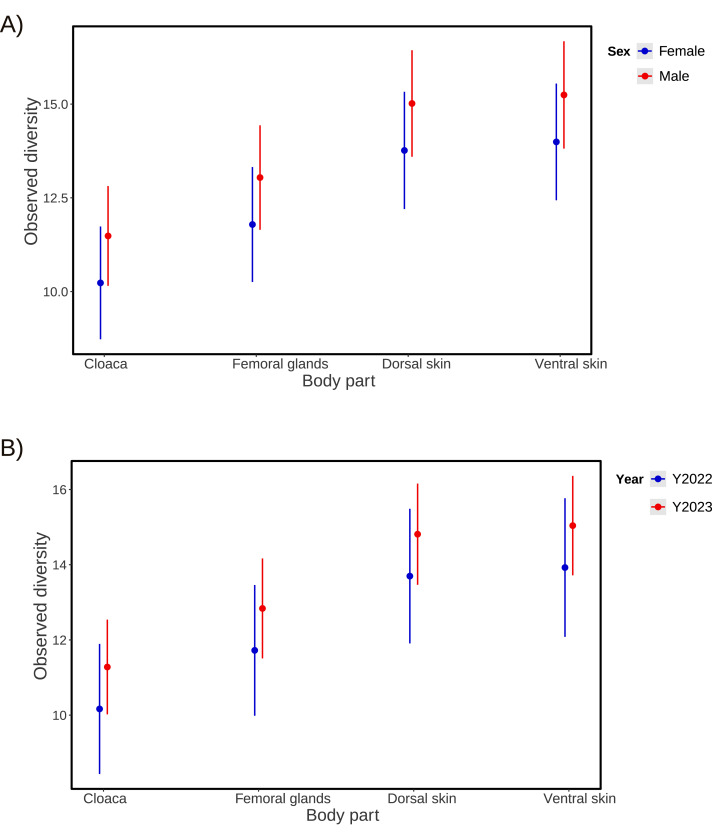
Results from microbial Observed diversity across body parts. Means and 95% confidence intervals of microbial Observed diversity across body parts (cloaca, femoral glands, dorsal skin, and ventral skin), obtained from a Generalized Linear Mixed Model, showing differences among them. Microbial diversity is shown for each sex (A) or each year (B) separately. *P*-values and other statistics are shown in Table 2.

**Figure 5:**
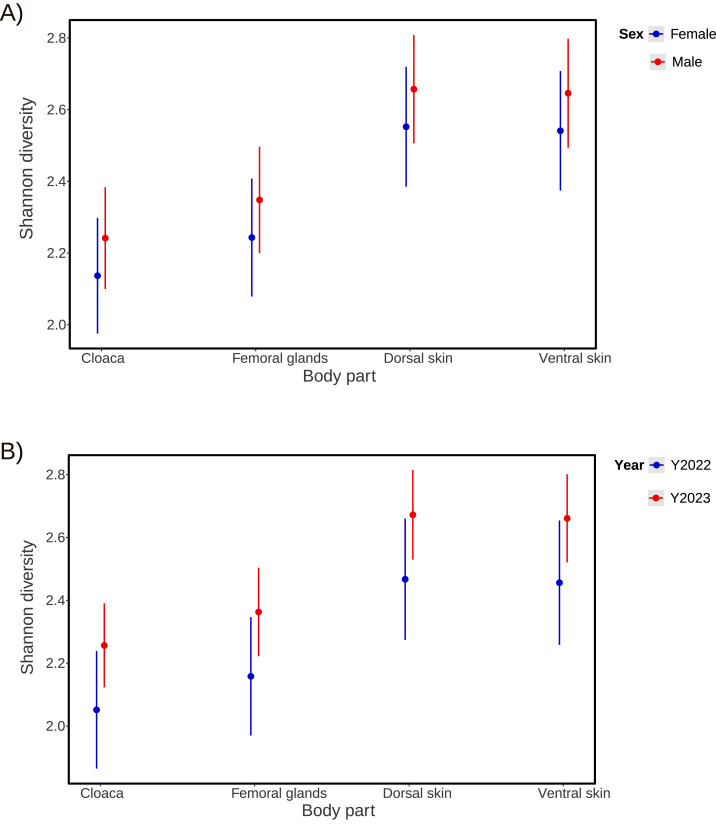
Results for microbial Shannon diversity across body parts. Means and 95% confidence intervals of microbial Shannon diversity across body parts (cloaca, femoral glands, dorsal skin, and ventral skin), obtained from a Generalized Linear Mixed Model, showing differences among them. Microbial diversity is shown for each sex (A) or each year (B) separately. *P*-values and other statistics are shown in Table 3.

There was a significantly greater mean for Observed microbial diversity in the femoral gland compared to the cloaca, but no significant difference based on the Shannon index was detected (Tables 2 and 3; Fig. 3). Dorsal and ventral skin samples had highly significantly greater microbial diversity than the cloaca, based on both Observed diversity (Table 2; Fig. 4) and the Shannon index (Table 3; Fig. 5). There was no significant sex difference in microbial diversity based on either Observed diversity (Table 2; Fig. 4) or Shannon index (Table 3; Fig. 5). Between years, we observed greater microbial diversity in 2023 than 2022 based on the Shannon index (Table 3; Fig. 5), but no difference was detected based on Observed diversity (Table 2; Fig. 4). In both models, the intercept represents the estimated microbial diversity for samples taken from the cloaca of female individuals in the reference year (*i.e.,* 2022). All reported coefficients for body part, sex, and year indicate deviations from this baseline.

**Table 2:** Differences observed in microbial diversity. Results of a Linear Mixed Model for differences in Observed microbial diversity as a function of body part, sex and year. Individual specimens nested within locations were included as random effects. The intercept represents the estimated microbial diversity for samples taken from the cloaca of female individuals in the reference year (*i.e.*, 2022). All reported coefficients for body part, sex, and year indicate deviations from this baseline.

** **	**Observed diversity**		
*Coefficient*	*Estimates*	95% CI	*P-value*
Intercept	9.44	7.64–11.23	<0.001
Part_(Femoral)_	1.56	0.07–3.04	0.040
Part_(SkinDorsal)_	3.53	2.02–5.04	<0.001
Part_(SkinVentral)_	3.76	2.24–5.28	<0.001
Sex_(Male)_	1.25	−0.36–2.87	0.129
Year_(2023)_	1.11	−0.67–2.90	0.220
**Random Effects**			
*σ* ^2^	19.45		
*τ*_00_ _Specimen∖Location_	5.49		
*τ*_00_ _Location_	0.00		
ICC	0.22		
N_Specimen_	77		
N_Location_	9		
Observations	266		
Marginal R^2^/Conditional R^2^	0.117/0.311		

**Table 3:** Differences of microbial diversity based on Shannon index. Results of a Linear Mixed Model for differences in the Shannon index of microbial diversity as a function of body part, sex and year. Individual specimens nested within locations were included as random effects. The intercept represents the estimated microbial diversity for samples taken from the cloaca of female individuals in the reference year (*i.e.*, 2022). All reported coefficients for body part, sex, and year indicate deviations from this baseline.

** **	**Shannon diversity**		
*Coefficient*	*Estimates*	95% CI	*P-value*
Intercept	1.99	1.80–2.19	<0.001
Part_(Femoral)_	0.11	−0.04–0.26	0.168
Part_(SkinDorsal)_	0.42	0.26–0.57	<0.001
Part_(SkinVentral)_	0.40	0.25–0.56	<0.001
Sex_(Male)_	0.10	−0.07–0.28	0.247
Year_(2023)_	0.20	0.01–0.40	0.041
**Random Effects**			
*σ* ^2^	0.20		
*τ*_00_ _Specimen∖Location_	0.08		
*τ*_00_ _Location_	0.00		
N_Specimen_	77		
N_Location_	9		
Observations	266		
Marginal R^2^/Conditional R^2^	0.198/0.384		

### Core microbiome and unique bacteria

**Figure 6:**
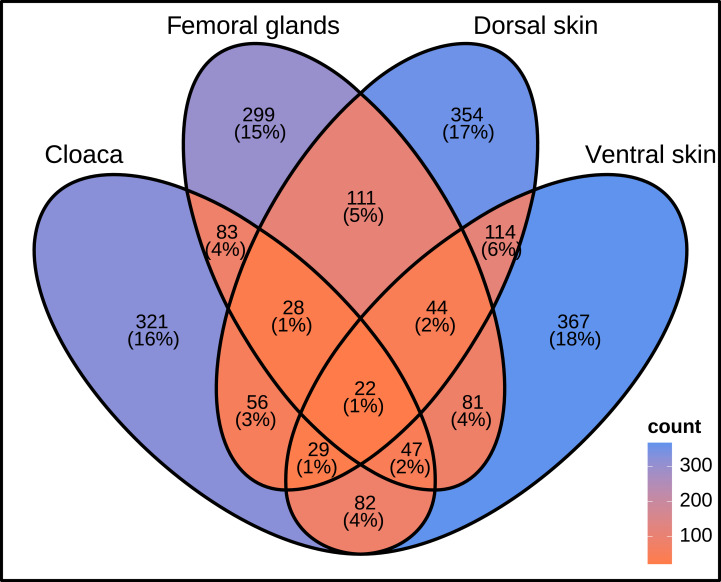
Venn Diagram and core microbiome. Number (and percentage) of shared and unique operational taxonomic units across the four body parts sampled in this study.

Considering all OTUs (2,038), only a small fraction (22) was shared by all tissues (Fig. 6). Therefore, the sand lizard core microbiome—resident microbiota shared across body parts—comprised only around 1% of overall microbial taxa. In contrast, the number of unique OTUs in each body part (*i.e.,* tissue-specific bacteria) represents a much higher portion of the total microbiome (around 15–18%). The number of unique OTUs showed a similar magnitude across body parts, ranging from a maximum of 367 in ventral skin, followed by 354 in dorsal skin, 321 in the cloaca, and 299 in femoral glands (Fig. 6).

### Sex variation in tissue-specific bacteria

**Figure 7:**
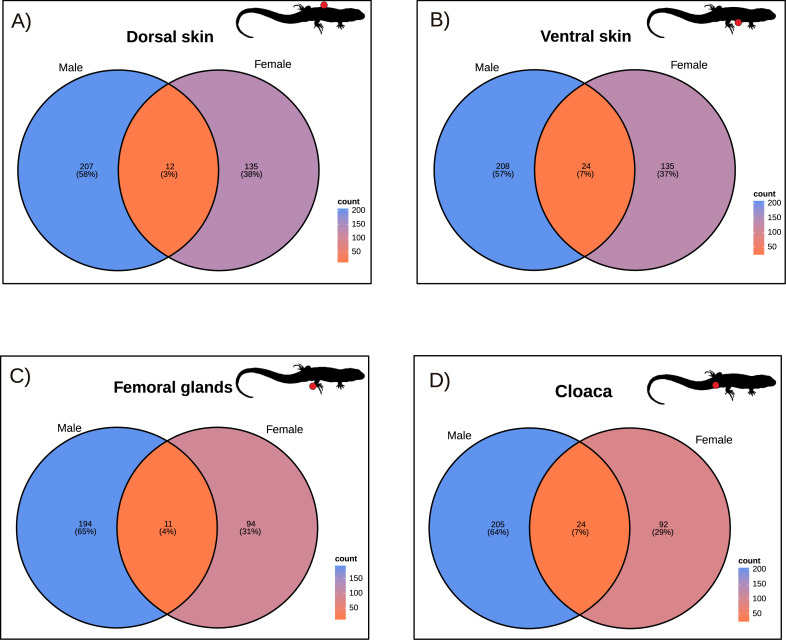
Sex related bacteria in different body parts. Sexual variation in tissue-specific bacteria (operational taxonomic units unique for a given body part). The number of shared and unique operational taxonomic units for males and females is shown. (A) dorsal skin; (B) ventral skin; (C) femoral glands; and (D) cloaca.

Tissue-specific bacteria showed little overlap in the number of shared bacteria between sexes (Fig. 7). Shared taxa between males and females only constituted around 3–7% of the microbial taxa. The number of shared OTUs was 24 in both ventral skin and cloaca, 12 in dorsal skin and 11 in femoral glands (see Fig. 7). We observed a general trend for males to have a larger number of unique OTUs (194 in femoral glands; 205 in cloaca; 207 in dorsal skin, and 208 in ventral skin) when compared to females (92 in cloaca; 94 in femoral glands, and 135 in both dorsal and ventral skin). However, there was no significant association between unique and shared OTUs between the sexes for dorsal skin (Fisher’s Exact Test, *P* = 0.390), ventral skin (*P* = 0.162), or femoral glands (*P* = 0.106), though there was for the cloaca (*P* = 0.013).

## Discussion

Our study provides a comprehensive characterization of the microbiome in sand lizards. We found out that microbial diversity changed pronouncedly across four body regions (cloaca, femoral glands, ventral skin, and dorsal skin). Microbial diversity was highest in the skin, both dorsal and ventral. The cloaca harboured a less diverse microbiome than any other region. Furthermore, microbial diversity differed across the year of sampling, indicating an important temporal effect on the lizard microbiome. However, we did not find an effect of sex on microbial diversity. Further analyses targeting unique and shared bacteria revealed that a core microbiome is constituted by a reduced number of taxa. In general terms, we found little overlap in the number of shared and unique OTUs between males and females when considering tissue-specific bacteria. However, a significant association between unique and shared OTUs between the sexes was found only in the cloaca region.

### Microbiome composition

In our study, most of the bacterial OTUs identified in sand lizards (*L. agilis*) belonged to Pseudomonadota (*i.e., Proteobacteria*) and Actinomycetota (*Actinobacteria*), while smaller proportions were classified as Bacteroidota (*Bacteroidetes*) and Bacillota (*Firmicutes*). Based on the most abundant OTUs, the cloaca and femoral glands were more similar to each other compared to both dorsal and ventral skin in sand lizards. We observed a striking variation in the relative abundance of some phyla in distinct body parts. For instance, dorsal and ventral skin were dominated by members of Bacillota and Bacteroidota. While these phyla were also among the most abundant in femoral glands and cloaca, members of Pseudomonadota and Campylobacterota were also observed. In the case of Campylobacterota, these bacteria were prominent in the cloaca, but this phylum was much underrepresented in the femoral glands and even absent in the skin (Fig. 2A). Interestingly, Campylobacterota are a group of Gram-negative bacteria that includes both free-living and pathogenic species posing a risk for human health (Gilbert et al., 2019). Whether Campylobacterota taxa present in sand lizards have a pathogenic effect deserves more attention in future research studies.

In general terms, the most common phyla present in sand lizard samples are usually found in other vertebrates. However, the abundance of predominant bacteria may vary across species and organs. For example, in Krefft’s river turtle (*Emydura macquarii krefftii*), Pseudomonadota and Bacteroidota were the most common phyla, but the amount of these taxa varied across samples originating from body internal (oral) and external (skin and shell) surfaces (McKnight et al., 2020). In another study focusing on the microbiome of cloaca, skin, feathers, as well as parts of nest material in two sympatric birds, the microbiome was dominated by Pseudomonadota, Acidobacteriota (Acidobacteria), Actinomycetota, Bacteroidota, and Bacillota, but the relative abundance of each phylum significantly varied among sample types (van Veelen, Falcao Salles & Tieleman, 2017). From a functional point of view, differences in microbiome composition may lead to differences in host metabolism (Rojo et al., 2017). Whether shifts in microbiome composition have an effect on sand lizard host metabolism remains to be investigated. In any case, our study indicates that a combination of ecological and environment-related factors specific to each body part may shape differences in major bacterial phyla.

### Microbial diversity, body regions and temporal variation

We found sharp differences in microbial diversity of distinct body parts of sand lizards. This result reflects the distinct microhabitat conditions intrinsic to each body region. The skin, being in direct contact with the external environment, showed the highest value of microbial richness compared to any other body part. Sand lizards use underground burrows and other refuges to protect themselves from unfavourable climatic conditions during the night (Blanke & Fearnley, 2015). Taking into account that microbial communities are ubiquitous in the soil (Nazir et al., 2024), it is likely that skin bacteria are picked up from contact with the surface of the soil. Similarly, exposure to other environmental agents, as well as contact between conspecifics during daily activities, might permit the colonization of a high diversity of environmental bacteria on the skin. As a result, skin-associated microbiota often reflect local abiotic and biotic conditions and display broad taxonomic diversity (Ross, Rodrigues Hoffmann & Neufeld, 2019). In contrast, internal or glandular regions such as the cloaca and femoral glands represent less accessible environments for extraneous bacteria. The micro-environment of these body parts may impose a strong selection for specific bacterial taxa, constraining the growth of some microbes, and thus reducing microbial diversity. For instance, the oily and hydrophobic environment of epidermal glands, such as human sebaceous glands, may promote colonization by lipophilic microorganisms but may inhibit the growth of pathogenic bacteria due to the acidic pH (Grice & Segre, 2011). In addition, antimicrobial agents might reduce bacterial diversity in the femoral glands and especially the cloaca. For example, antimicrobial peptides are secreted by femoral glands in marine iguanas *Amblyrhynchus cristatus* (Tellkamp et al., 2020), but it is not known whether these are present in glandular secretions of other lizards. In the case of the cloaca, antimicrobial properties were detected in olive ridley sea turtles, *Lepidochelys olivacea* (Praja et al., 2021). Therefore, a possible hypothesis could be that the cloaca of sand lizards secretes antimicrobial compounds as a mechanism to cope with pathogenic bacteria and infectious agents, a fact that could explain the observed low microbial diversity in this body region.

We found that microbial diversity (Shannon index) changed significantly between years suggesting that sand lizard microbiomes are dynamic rather than fixed. Longitudinal studies have demonstrated that microbial communities are highly variable across individuals and time scales in humans and other primates (Flores et al., 2014; Muletz-Wolz et al., 2019; Fenn et al., 2023). In wild animals, longitudinal studies are scarce, and most of the research has focused on the mammalian gut microbiome, for which microbiome stability could depend on host ecological context (Marsh, Bearhop & Harrison, 2024). In addition, seasonal or inter-annual changes in diet, climate, and host physiology may lead to recurrent reorganizations of microbial communities, as documented in rodents and primates (Marsh et al., 2022; Fan et al., 2022; Baniel et al., 2021). However, the stability of microbial communities may also vary across species—for instance, the cloacal microbiome of striped plateau lizards changed little between years (Bunker, Arnold & Weiss, 2022). Therefore, microbiome temporal plasticity might be site- and species-dependent, and probably influenced by host behaviour, life history, and environmental variability. In any case, our results indicate an important inter-year temporal effect on sand lizard microbial diversity, supporting the concept of a highly dynamic microbiome in wild hosts. Our study highlights the need to consider seasonal effects as a relevant factor in future studies on reptile microbiome.

### Core microbiome and sexual patterns in tissue-specific bacteria

Only a minor part (around 1%) of all microbes was shared across all body parts, meaning that the core microbiome in sand lizards consisted of very few microbial taxa. Similar patterns have been reported in other vertebrates, where shared or “core” taxa across tissues usually represent only 1–5% of the total microbial diversity (Ursell et al., 2012). In humans, the Human Microbiome Project found less than 2% of OTUs consistently present within a single body site and almost no overlap between different body regions (Huse et al., 2012). Likewise, in marine mammals, microbial communities from the mouth, stomach, and rectum showed *“surprisingly little overlap”* (Bik et al., 2016). In the gilt-head sea bream, *Sparus aurata*—a marine teleost fish—the gut shared fewer than 13% of ASVs with the skin or gills (Quero et al., 2023). Studies on passerine birds also showed that the cloaca, skin, and feathers shared only a small fraction of bacterial taxa (van Veelen, Falcao Salles & Tieleman, 2017). Our results are also consistent with other lizards (*e.g.*, genus *Podarcis* and *Sceloporus*; (Bunker, Arnold & Weiss, 2022; Alemany et al., 2023)) in which core microbiomes are constituted by a small number of taxa. Therefore, we can conclude that sand lizard microbiomes are highly compartmentalized. Previous research has suggested that host-associated core microbiomes are important from a functional point of view, playing key roles in host physiology, immunity, or homeostasis (Furusawa et al., 2013; Atarashi et al., 2013). For example, a core human gut microbiome is involved in host metabolism and may affect individual physiological state (Turnbaugh & Gordon, 2009). Whether the core microbiome plays a role in sand lizard physiological state or health condition remains unknown. However, our results revealed that there is limited overlap in the microbiota harboured by distinct body regions, suggesting that any potential functional role of the core microbiome in sand lizards is restricted to just a few bacteria.

The number of unique microbial taxa for each body part (tissue-specific bacteria) was much higher than the number of OTUs constituting the core microbiome. Approximately 15–18% of the OTUs were specific for each body part (see Fig. 6). The low overlap between the skin, femoral glands, and cloaca indicates that each body part harbours a dynamic microbiome rather than a stable set of host-associated bacteria. This finding suggests that environmental factors and transient colonization are more important than long-term coevolution for the establishment of microbial communities in sand lizards.

### Sexual variation in tissue-specific bacteria and chemical communication

Sex-related differences in microbial diversity have been previously reported in many animals (Levin et al., 2016; Wang et al., 2020; Xu & Zhang, 2021). Many bacterial taxa produce volatile compounds that are released to the environment (Schulz & Dickschat, 2007). Therefore, one possible scenario would be that sexual differences in microbial diversity may lead to distinct odoriferous profiles emitted by males and females that could be potentially used for social communication. However, the lack of sexual differences in microbial diversity in our study does not lend support to this hypothesis in sand lizards and instead suggests that more diverse microbiomes do not necessarily contribute to chemical signalling in this species.

As an alternative to this hypothesis, it could be that pheromonal production is related to just a few bacterial taxa. Interestingly, we found a minimal overlap (just 3–7%) between males and females in the number of shared OTUs for tissue-specific bacteria (see Fig. 7). Therefore, our results give preliminary support for this alternative hypothesis. In this hypothetical scenario, chemical signals could be produced by a restricted number of bacteria, such as tissue-specific taxa. Indeed, some specific bacterial taxa have been targeted as primary sources of chemosignals in vertebrate hosts. For instance, Brunetti et al. (2019) found that a bacterial strain of the genus *Pseudomonas* synthesizes methoxypyrazines—a specific class of nitrogen-containing compounds—in the skin of tree frogs (*Boana prasina*). In birds, the amount of some ketones—a type of organic compound—released by the uropygial glands was related to the abundance of certain bacterial OTUs (Whittaker et al., 2019). We speculate that a similar mechanism could be possible for the production of glandular lipophilic compounds in sand lizards (Ibáñez et al., 2025).

A low overlap in tissue-specific bacteria between sexes was observed for all body parts, and even significant differences were only detected in the cloaca. This result, together with a reduced microbial diversity detected in the cloaca—the lowest diversity among all body parts—indicates high specialization of the bacterial communities populating this organ. Previous findings in *S. virgatus* have shown that cloacal microbiomes are involved in the transference of beneficial microbes during oviposition, showing the uniqueness of these microbial communities (Bunker, Martin & Weiss, 2022; Bunker et al., 2021; Bunker, Arnold & Weiss, 2022). Indeed, this specialization could be related to the cloaca’s reproductive and excretory roles. In addition, a potential involvement in chemical communication of the cloaca could be mediated by microbial metabolites. For example, previous results in the fossorial amphisbaenian *Trogonophis wiegmanni* revealed that cloacal products are rich in lipophilic compounds, including intersexual differences in some compounds, suggesting a potential involvement in intraspecific communication (Martín et al., 2023). Although the chemical composition of the cloaca remains unstudied for most reptiles, our results indicate that sand lizard cloacal microbial communities are highly specific, with little overlap between sexes, giving promising avenues for further research on the interplay between cloacal microbiome and intraspecific communication.

Finally, we would like to highlight that a large portion of the bacterial OTUs included in our analysis could not be assigned to a given taxonomic lineage. While our strict filtering steps and statistical analysis provide a robust comparison of lizard microbial diversity and composition across body parts, sexes and years, future studies should focus on a more in-depth taxonomic characterization of unassigned bacteria.

## Conclusions

Our study revealed that sand lizards harbour rich and diverse microbial communities, pointing out that lizard microbiomes are highly dynamic, showing temporal variation and striking differences across the body, likely due to the microhabitat conditions. Microbial diversity was higher in dorsal and ventral skin, while femoral glands and cloaca harbor less diverse microbiomes. Sand lizards had an extremely small core microbiome (∼1% of shared taxa) as well as high tissue specificity, emphasizing that each body region provides a unique microbial niche, with limited overlap among tissues. Despite the fact that microbial diversity did not differ between males and females, sex variation was restricted to a smaller number of microbes (*i.e.,* tissue-specific bacteria). This pattern was especially evident in the cloacal region. Thus, the cloacal microbiome appeared highly specialized as demonstrated by its low microbial diversity and marked sexual difference. Our results give preliminary support to the hypothesis that the potential role of microbiome on chemical intraspecific communication could be related to a small number of unique microbial taxa rather than overall patterns of microbial diversity—a hypothesis that needs to be tested in future studies. Functional and experimental approaches to the sand lizard microbiome are necessary to fully understand how chemical communication is related to host-associated microbiomes.

##  Supplemental Information

10.7717/peerj.21061/supp-1 Supplemental Information 1 Filtering of Rare Operational Taxonomic Units Based on Density Distribution of Log-Transformed Abundances Density plot of log-transformed Operational Taxonomic Unit abundances used to identify and remove low-abundance taxa. The vertical line marks the first local minimum, applied as a threshold to exclude rare Operational Taxonomic Units from downstream analyses.

10.7717/peerj.21061/supp-2 Supplemental Information 2 Taxonomy classification of sequences Percentage of Operational Taxonomic Units classified in different bacterial phyla and main categories based on the results from the blastn alignment. Results are summarised for all taxa (left) according to the legend. Minor phyla are zoomed in (right) for a better visualization.

10.7717/peerj.21061/supp-3 Supplemental Information 3 Composition of lizard samples and controls Most abundant phyla (top 15 Operational Taxonomic Units, from Dataset-2) for sand lizard body parts and control samples. Note the striking differences between controls and lizard samples.

10.7717/peerj.21061/supp-4 Supplemental Information 4 PCR conditions Conditions and primers for the PCR used in this study .

10.7717/peerj.21061/supp-5 Supplemental Information 5 Comparison of the number of unassigned (‘under threshold’) bacteria using two different thresholds Preliminary analysis showing the number of unassigned (‘under threshold’) bacteria using two differed thresholds in a subset of the samples (N=75; cloaca region swabs). A threshold of 60% was selected as optimal due its greater performance in bacterial taxonomic assignment.

## Additional Information and Declarations

### Field Study Permissions

The following information was supplied relating to field study approvals (*i.e.*, approving body and any reference numbers): The Directorate for Environmental Protection in Łódź (WPN.6401.10.2022.BWo) and the General Environmental Protection Directorate, Poland (DZP-WG.6401.116.2022.TŁ).

### Data Availability

The following information was supplied regarding data availability: The sequence data is available at NCBI SRA: PRJNA1355145.
