# Supplementary figures and images for: Universes within universes: microbiome diversity associated with different body parts of the sand lizard (Lacerta agilis)
Source: PeerJ. 2026 May 1;14:e21061. doi: 10.7717/peerj.21061 (PMC13138299; doi:10.7717/peerj.21061)

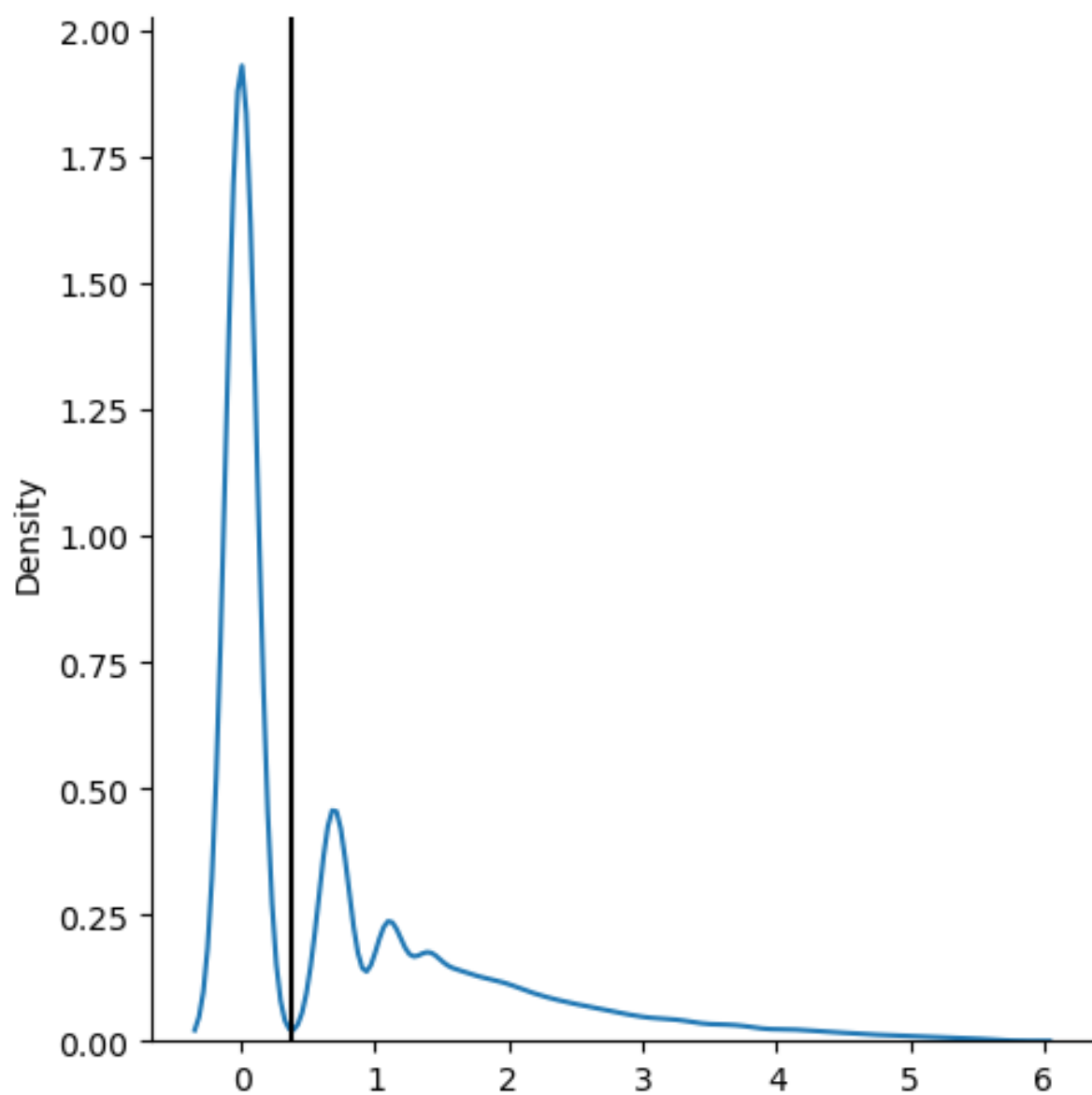

Supplement: Supplemental Information 1 — Density plot of log-transformed Operational Taxonomic Unit abundances used to identify and remove low-abundance taxa. The vertical line marks the first local minimum, applied as a threshold to exclude rare Operational Taxonomic Units from downstream analyses. [file peerj-14-21061-s001.pdf]

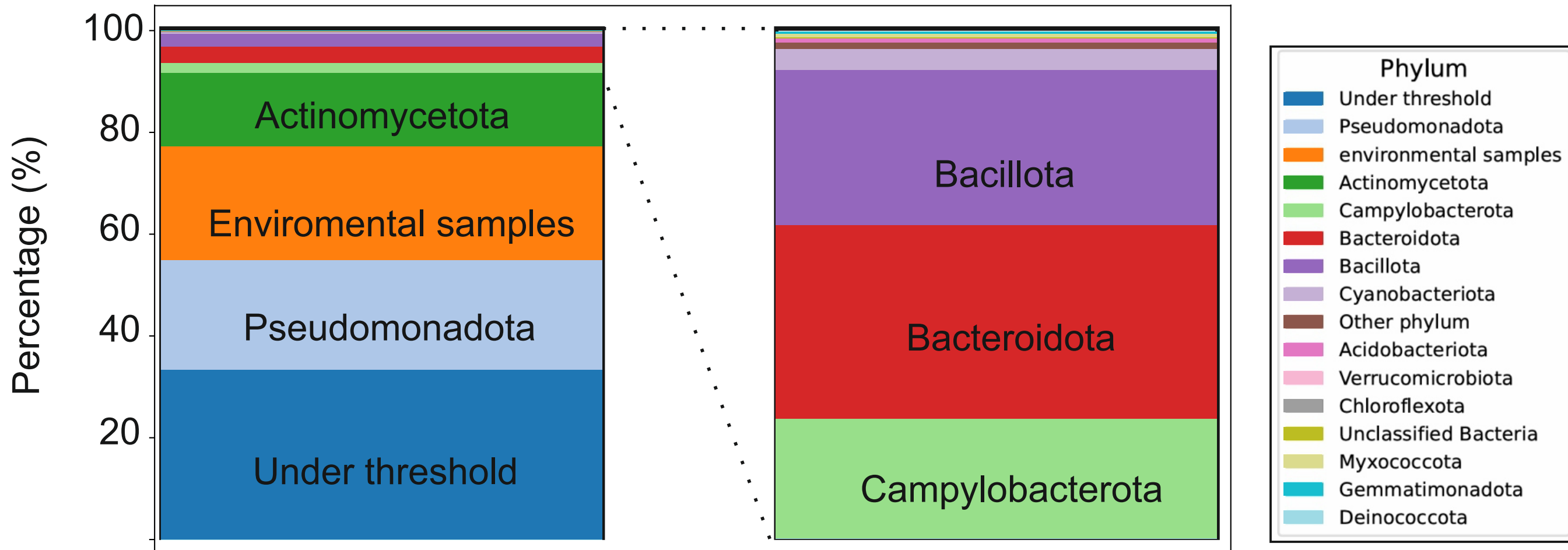

Supplement: Supplemental Information 2 — Percentage of Operational Taxonomic Units classified in different bacterial phyla and main categories based on the results from the blastn alignment. Results are summarised for all taxa (left) according to the legend. Minor phyla are zoomed in (right) for a better visualization. [file peerj-14-21061-s002.pdf]

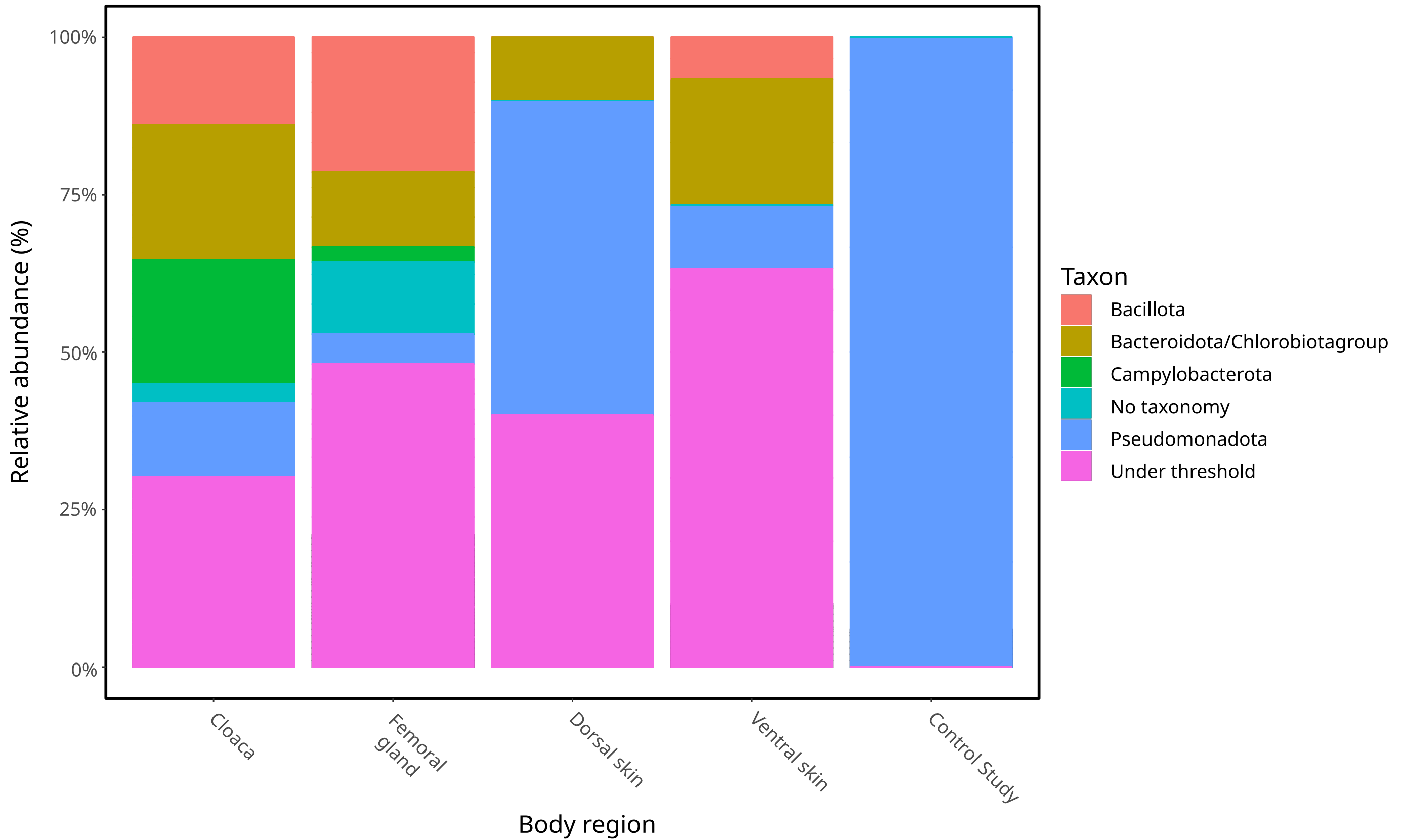

Supplement: Supplemental Information 3 — Most abundant phyla (top 15 Operational Taxonomic Units, from Dataset-2) for sand lizard body parts and control samples. Note the striking differences between controls and lizard samples. [file peerj-14-21061-s003.pdf]
