# Supplementary material for: Universes within universes: microbiome diversity associated with different body parts of the sand lizard (Lacerta agilis)
Source: PeerJ. 2026 May 1;14:e21061. doi: 10.7717/peerj.21061 (PMC13138299; doi:10.7717/peerj.21061)
Supplement: Supplemental Information 4 — Conditions and primers for the PCR used in this study . [file peerj-14-21061-s004.docx]

| Gene | Region | Name | Sequences (5’-3’) | AT  (^o^C) | Product size (bp) | References |
| --- | --- | --- | --- | --- | --- | --- |
| 16S rRNA | V3-V4 | 341f | CCTACGGGNGGCWGCAG | 55 | 444 | Klindworth et al., 2013 |
|  |  | 785r | GACTACHVGGGTATCTAATCC |  |  |  |
