# Supplementary material for: Universes within universes: microbiome diversity associated with different body parts of the sand lizard (Lacerta agilis)
Source: PeerJ. 2026 May 1;14:e21061. doi: 10.7717/peerj.21061 (PMC13138299; doi:10.7717/peerj.21061)
Supplement: Supplemental Information 5 — Preliminary analysis showing the number of unassigned (‘under threshold’) bacteria using two differed thresholds in a subset of the samples (N=75; cloaca region swabs). A threshold of 60% was selected as optimal due its greater performance in bacterial taxonomic assignment. [file peerj-14-21061-s005.docx]

| **Level assessed** | **60 % threshold** | **70% threshold** |
| --- | --- | --- |
| **Class** | 1924 | 3697 |
| **Order** | 2062 | 3791 |
| **Family** | 2259 | 3963 |
